# Prevalence and trends in long-term survivors of early-onset vs. late-onset cancer: a serial cross-sectional study

**DOI:** 10.3389/fpubh.2026.1714359

**Published:** 2026-01-30

**Authors:** Qiang Yin, Run Xu, Dongmei Wang, Jian Li

**Affiliations:** Department of General Surgery, The Third Hospital of Mianyang, Sichuan Mental Health Center, Mianyang, China

**Keywords:** early-onset cancer, late-onset cancer, prevalence, survivorship, temporal trends

## Abstract

**Background:**

With increasing global cancer survivorship, understanding differential trends by age at diagnosis is crucial for developing targeted care strategies. This study examines 27-year trends in early-onset (20–49 years) vs. late-onset (≥50 years) cancer survivorship in the US population.

**Methods:**

We analyzed nationally representative data from the National Health Interview Survey (1997–2023), identifying adults surviving ≥5 years post-diagnosis. Weighted prevalence estimates were calculated, and temporal trends were analyzed using Joinpoint regression to compute average annual percentage changes (AAPCs) with 95% confidence intervals (CIs).

**Results:**

Women represented 72.7% of early-onset cancer survivors, compared to 51.5% of late-onset cancer survivors. From 1997 to 2023, both prevalence of early-onset (AAPC 1.7, 95% CI: 1.4 to 2.0) and late-onset (AAPC 1.9, 95% CI: 1.7 to 2.1) cancer survivors increased significantly. The prevalence of male early-onset cancer survivors rose the most sharply (AAPC 3.1, 95% CI: 2.5 to 3.8), compared to prevalence of late-onset (AAPC 1.9, 95% CI: 1.6 to 2.3) or female cancer survivors (AAPC early-onset 1.3, 95% CI: 1.0 to 1.6; late-onset 1.7, 95% CI: 1.5 to 2.0). College-educated individuals had higher baseline prevalence and faster growth. Similarly, high-income groups showed elevated prevalence (early-onset 2.65%; late-onset 8.22%) and the most rapid increase was found in late-onset cancer survivors (AAPC 3.2, 95% CI: 2.5 to 3.9).

**Conclusion:**

These findings demonstrate universal increases in cancer survivorship with distinct socioeconomic and gender patterns, highlighting the need for tailored survivorship programs and targeted policies to address emerging disparities in long-term cancer care.

## 1 Introduction

The global burden of cancer has traditionally been assessed through metrics such as mortality, incidence, or disability-adjusted life years, which dominate research agendas and public health policies (1–3). While these indicators provide crucial insights into disease patterns and treatment efficacy, they fail to account for the growing population of long-term cancer survivors, who now constitute a significant proportion of the global cancer burden. GLOBOCAN 2022 evaluated cancer survival only within 5 years of diagnosis (2). Although the Global Burden of Disease (GBD) 2021 reported cancer prevalence, results stratified by survival duration were unavailable (1). The American Cancer Society (ACS) and the National Cancer Institute (NCI) triennially report cancer survivorship statistics, estimating prevalence by years since diagnosis; however, data on temporal trends and differences between early-onset (diagnosed at ages 20–49) and late-onset (diagnosed at age 50 or older) cancer survivors are lacking (4).

This knowledge gap is particularly critical when examining differences between early-onset and late-onset cancer survivors. Early-onset survivors face unique challenges, including elevated risks of treatment-related chronic conditions, secondary malignancies, and premature mortality from non-cancer causes (5–7). Emerging evidence suggest that they may experience accelerated aging phenotypes and higher risks of severe frailty compared to the general population (8, 9). Conversely, older survivors often contend with complex interactions between cancer sequelae and age-related multimorbidity, yet their long-term outcomes remain poorly characterized (10, 11). These distinct risk profiles underscore the urgent need for age-specific survivorship care approaches that account for varying health trajectories across the lifespan.

This study addresses these gaps by leveraging nationally representative data from the National Health Interview Survey (NHIS) to analyze the prevalence of long-term cancer survivors comprehensively. Shifting focus from traditional mortality metrics to prevalence-based assessments, the study provides novel insights into the evolving epidemiology of cancer survivorship. The findings will inform nuanced approaches to long-term survivorship care planning and resource allocation, particularly for vulnerable subgroups at elevated risk of long-term complications.

## 2 Methods

### 2.1 Study design and data source

This study analyzed data from the NHIS, an annual nationally representative cross-sectional survey conducted by the National Center for Health Statistics. The NHIS employs a complex, multistage probability sampling design to collect health-related data from the civilian, non-institutionalized US population. The survey includes self-reported demographic, socioeconomic, and health-related variables, with cancer history, age at diagnosis, and survival duration extracted from the Sample Adult, Household, and Family files. Publicly available NHIS documentation and datasets can be accessed at: https://www.cdc.gov/nchs/nhis/documentation/index.html. To assess trends in long-term cancer survivorship, we included participants aged ≥25 years from 1997 to 2023. For contemporary prevalence estimates, we restricted the analysis to the most recent 5 years (2019–2023).

### 2.2 Definition of cancer survivors

During the interviews, participants self-reported their cancer history, including the type of cancer and their age at diagnosis. Based on this information, individuals were classified into distinct groups: early-onset cancer survivors were individuals whose cancers were diagnosed between ages 20–49 years; late-onset cancer survivors were individuals whose cancers were diagnosed at ≥50 years. In this study, we only focused on long-term survivors who have survived for at least 5 years post-diagnosis, a threshold generally considered indicative of cancer cure. It is worth noting that definitions of early-onset cancer vary across studies, with some using age thresholds of <45 or <40 years for specific cancer types. In this study, we adopted the 20–49 and ≥50 years classification to maintain consistency with existing literature and facilitate comparisons across studies while maintaining methodological simplicity, and to ensure sufficient sample size for stable trend analyses across a wide range of cancers (12). While this broad categorization may not capture finer biological or clinical distinctions within age groups, it provides a pragmatic framework for population-level comparisons. In this study, non-melanoma skin cancers were excluded for analysis. Due to the limited number of cases for certain cancer types, cancers were categorized into the following groups: hematological cancer (blood, leukemia and lymphoma), breast cancer, gynecological cancer (cervical, ovarian and uterine), colorectal cancer (colon and rectal), non-colorectal gastrointestinal cancer (esophageal, gallbladder, liver, pancreatic and stomach), head and neck cancer (larynx-trachea, mouth, tongue, lip and throat-pharynx), thyroid cancer, urinary cancer (bladder and kidney), male-specific cancer (prostate and testicular), lung cancer, and others.

### 2.3 Assessment of sociodemographic characteristics

The study incorporated key sociodemographic variables known to influence cancer outcomes, including sex, age, race/ethnicity, education level, and family economic status. Race and ethnicity were categorized as non-Hispanic White people, non-Hispanic Black people, Hispanic, and others based on self-reported data from standardized questionnaires. Educational attainment was classified into less than high school, high school diploma or equivalent, and college degree or higher, maintaining consistency with previous national health surveys (13, 14). Family economic status was assessed using the Poverty Income Ratio (PIR; also known as the family income-to-poverty ratio), with participants grouped into four categories (<1.00, 1.00–1.99, 2.00–3.99, ≥4.00), reflecting both federal poverty guidelines and relative income distribution patterns (13). These sociodemographic factors were selected for analysis due to their well-documented associations with healthcare access, treatment disparities, and long-term survival outcomes in cancer populations.

### 2.4 Statistical analysis

The statistical analysis employed weighted methods to account for the complex survey design of the NHIS, ensuring nationally representative estimates. Prevalence estimates with 95% confidence intervals (CIs) were calculated for both early-onset (in populations aged 25 years or older) and late-onset (in populations aged 55 years or older) long-term cancer survivors. For participants reporting multiple cancers, each cancer was treated as a separate observation due to potential differences in long-term effects. Temporal trends from 1997 to 2023 were analyzed using Joinpoint regression to estimate average annual percentage changes (AAPCs) and 95% CIs, which identified significant shifts in prevalence trajectories while adjusting for autocorrelation. Subgroup analyses were stratified by sex, race and ethnicity, education level, PIR, cancer type, survival time (5–9, 10–14, 15–19, 20–24 and ≥25 years), age at screening (25–49, 50–69, and ≥75 years). All analyses incorporated sampling weights and accounted for clustering and stratification in the NHIS design using IBM SPSS Statistics 25 (IBM Co., Armonk, NY, United States), STATA 14.0 (Stata Corporation, Texas, United States) and the Joinpoint Regression Program 4.9.0.0 (National Cancer Institute, Rockville, MD, United States). The statistical significance was set at two-tailed *p* < 0.05. In this study, AAPC with a 95% CI greater than 0 indicates that the prevalence has a significant increasing trend, whereas a value less than 0 indicates a significant decreasing trend; otherwise, the prevalence is considered stable or volatile. Non-overlapping 95% CIs were considered statistically significant when comparing prevalence and changing trends between early-onset and late-onset cancer survivors and across subgroups.

## 3 Results

### 3.1 Participant characteristics

**Figure 1:**
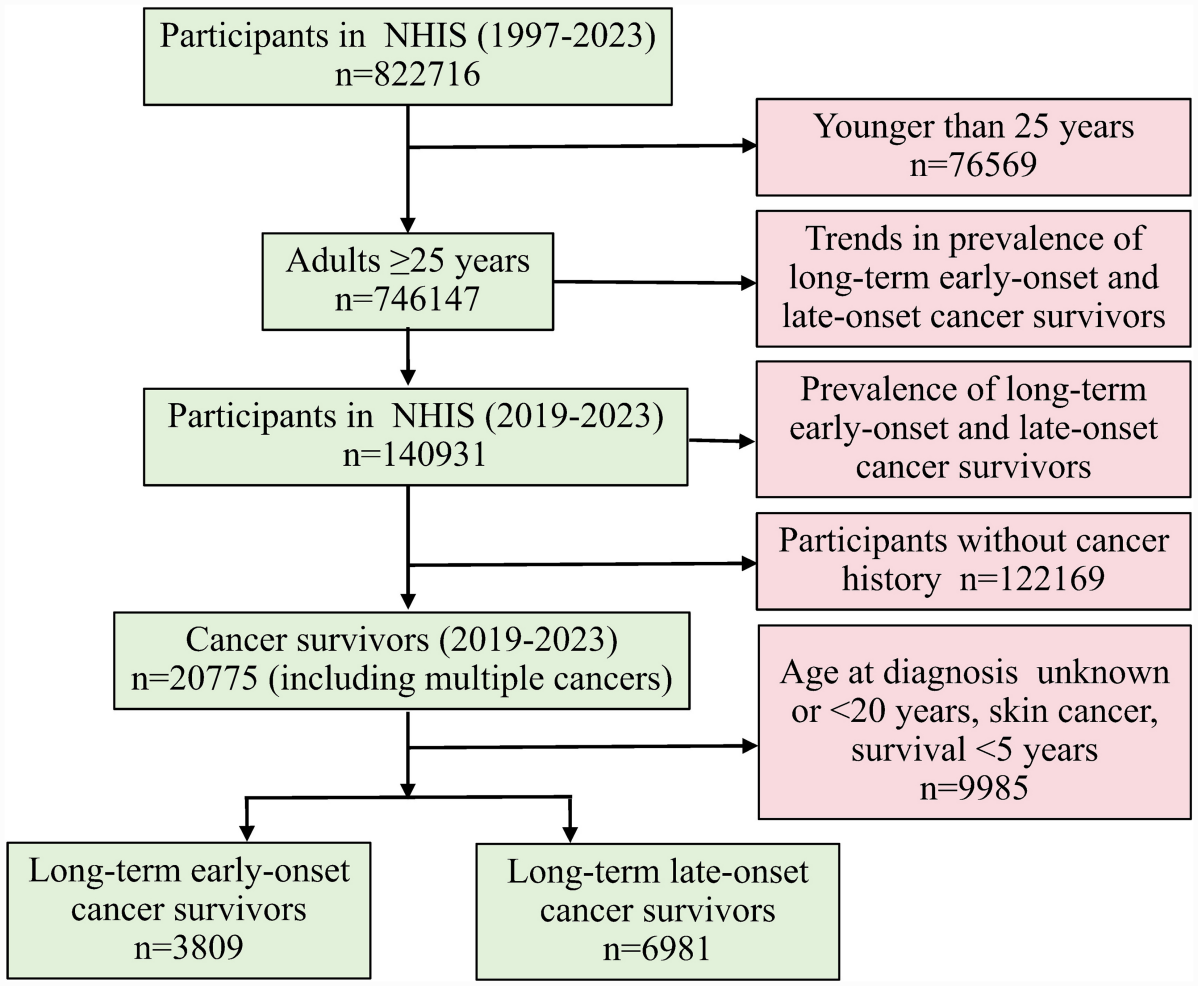
Flowchart of the participants identified and extracted from the NHIS. NHIS, National Health Interview Survey.

From 1997 to 2023, our analysis included 822716 adults in health interviews. After applying inclusion criteria, 746147 adults aged ≥25 years were available for trend analysis, representing a nationally weighted population with a mean age of 50.18 ± 0.05 years. The cohort demonstrated balanced sex distribution (47.96% men, 95% CI: 47.80 to 48.12%; 52.04% women, 95% CI: 51.88 to 52.20%), with the majority aged 25 to 49 years (51.68, 95% CI: 51.39 to 51.97%). Socioeconomic characteristics revealed 58.41% (95% CI: 57.98 to 58.84%) attained college-level education or higher, while 37.16% (95% CI: 36.69 to 37.64%) had a PIR higher than 4.0. Racial/ethnic composition showed predominance of non-Hispanic White people (66.50, 95% CI: 65.84 to 67.15%), followed by non-Hispanic Black people (11.03, 95% CI: 10.65 to 11.43%) and Hispanics (13.18, 95% CI: 12.69 to 13.68%). For contemporary prevalence estimates, we analyzed 140931 participants from 2019 to 2023 (Figure 1).

### 3.2 Overall prevalence differences

**Figure 2:**
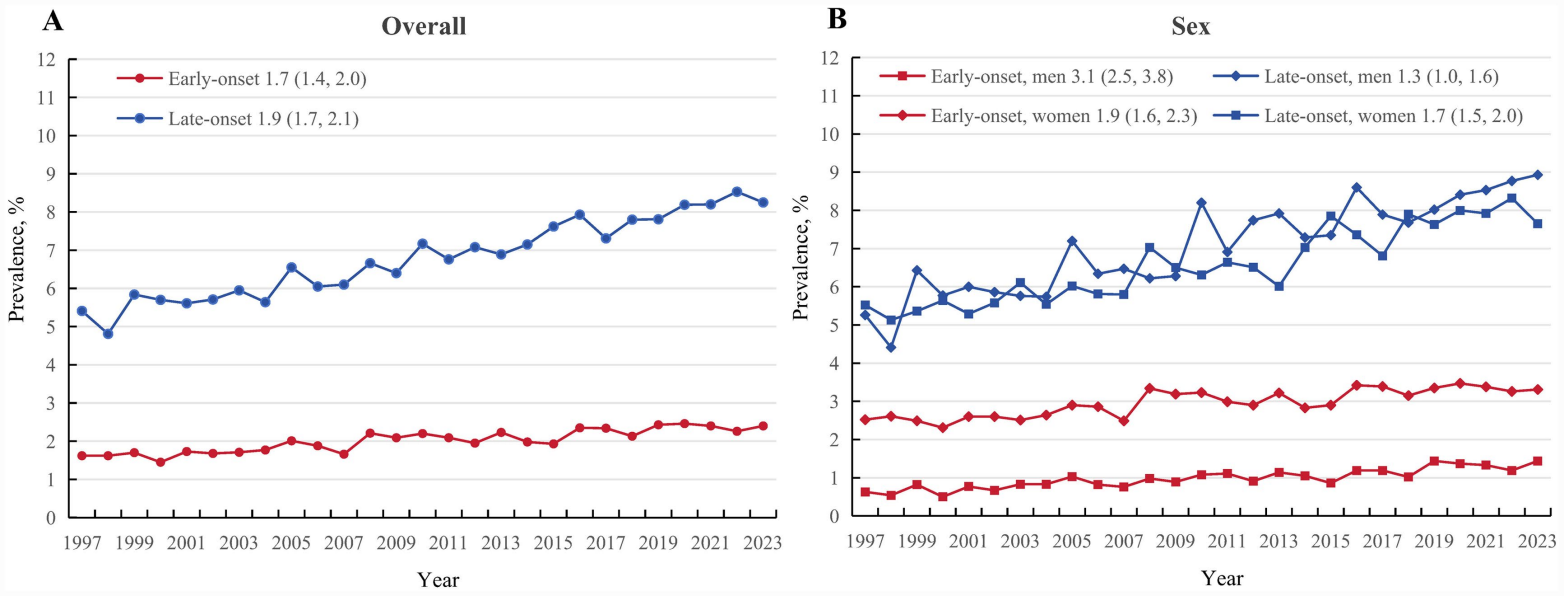
Trends in the prevalence of long-term cancer survivors among US adults overall **(A)** and by sex **(B)**, 1997 to 2023.

During 2019 to 2023, we identified 10790 long-term adult survivors of cancer, representing a prevalence of 6.01% (95% CI: 5.84 to 6.18%). Early-onset cancer survivors accounted for 39.78% (95% CI: 38.61 to 40.96%), while late-onset cancer survivors comprised 60.22% (95% CI: 59.04 to 61.39%). Early-onset cancer survivors showed significantly lower prevalence (2.39, 95% CI: 2.30 to 2.49%) compared to late-onset cancer survivors (8.20, 95% CI: 7.95 to 8.45%; Table 1). Over the 27-year study period, the prevalence of early-onset cancer survivors increased from 1.62% (95% CI: 1.49 to 1.78%) in 1997 to 2.40% (95% CI: 2.20 to 2.62%) in 2023, with AAPC of 1.7 (95% CI: 1.4 to 2.0), while the prevalence of late-onset cancer survivors increased from 5.41% (95% CI: 4.96 to 5.89%) to 8.25% (95% CI: 7.76 to 8.77%), representing a 1.9% (95% CI: 1.7 to 2.1%) annual change (Figure 2; Table 2).

**Table 1:** Differences in prevalence between long-term survivors of early-onset and late-onset cancer, 2019–2023.

Subgroups	Early-onset cancer		Late-onset cancer		*p* value
	Cases (weighted %)	Prevalence, % (95% CI)	Cases (weighted %)	Prevalence, % (95% CI)	
Overall	3809 (39.8)	2.39 (2.30, 2.49)	6981 (60.2)	8.20 (7.95, 8.45)	
Sex					<0.001
Men	914 (27.3)	1.36 (1.25, 1.47)	3200 (48.5)	8.54 (8.17, 8.92)	
Women	2895 (72.7)	3.36 (3.20, 3.52)	3781 (51.5)	7.91 (7.60, 8.22)	
Age at interview, years					<0.001
25–49	775 (26.4)	1.34 (1.23, 1.47)	Not applicable	Not applicable	
50–69	2074 (55.2)	3.69 (3.50, 3.90)	1808 (29.9)	4.05 (3.82, 4.29)	
70–85	954 (18.5)	2.56 (2.37, 2.76)	5159 (70.1)	14.68 (14.20, 15.17)	
Education					<0.001
Less than high school	287 (9.0)	2.00 (1.71, 2.33)	568 (10.3)	6.58 (5.96, 7.27)	
High school	849 (24.2)	2.18 (1.99, 2.38)	1759 (27.9)	7.93 (7.51, 8.38)	
College or above	2660 (66.7)	2.56 (2.44, 2.68)	4624 (61.8)	8.73 (8.42, 9.05)	
PIR					<0.001
<1.00	451 (11.1)	2.83 (2.49, 3.22)	439 (5.9)	5.45 (4.82, 6.15)	
1.00–1.99	663 (17.2)	2.34 (2.11, 2.59)	1313 (18.4)	8.24 (7.71, 8.81)	
2.00–3.99	1086 (28.3)	2.27 (2.09, 2.45)	2288 (33.0)	8.95 (8.50, 9.42)	
≥4.00	1609 (43.4)	2.40 (2.26, 2.54)	2941 (42.6)	8.22 (7.88, 8.58)	
Race					<0.001
Non-Hispanic White people	3113 (79.7)	2.98 (2.85, 3.11)	5971 (83.2)	9.43 (9.14, 9.73)	
Non-Hispanic Black people	261 (7.0)	1.46 (1.25, 1.69)	485 (7.6)	6.08 (5.45, 6.79)	
Hispanic	272 (8.5)	1.27 (1.07, 1.50)	309 (5.6)	4.29 (3.75, 4.90)	
Others	163 (4.7)	1.33 (1.07, 1.65)	216 (3.6)	4.42 (3.71, 5.25)	
Survival, years					<0.001
5–9	576 (18.5)	0.44 (0.40, 0.49)	2711 (40.8)	3.34 (3.19, 3.50)	
10–14	569 (17.9)	0.43 (0.39, 0.48)	1982 (29.1)	2.39 (2.27, 2.51)	
15–19	517 (15.5)	0.37 (0.33, 0.41)	1118 (15.1)	1.24 (1.15, 1.33)	
20–24	551 (14.3)	0.34 (0.31, 0.37)	717 (9.3)	0.76 (0.69, 0.83)	
≥25	1596 (33.9)	0.81 (0.76, 0.86)	453 (5.7)	0.47 (0.42, 0.53)	
Cancer types					<0.001
Hematological	217 (6.5)	0.15 (0.13, 0.18)	343 (4.8)	0.39 (0.34, 0.45)	
Breast	830 (19.1)	0.87 (0.80, 0.95)	1746 (23.7)	3.59 (3.39, 3.81)	
Gynecological	1037 (26.9)	1.24 (1.14, 1.35)	402 (5.6)	0.86 (0.76, 0.97)	
CRC	145 (3.7)	0.09 (0.07, 0.11)	539 (7.8)	0.64 (0.58, 0.71)	
NonCRC GI	38 (1.1)	0.08 (0.06, 0.10)	136 (2.2)	0.18 (0.14, 0.22)	
Head and neck	45 (1.3)	0.03 (0.02, 0.04)	119 (1.8)	0.15 (0.12, 0.18)	
Thyroid	256 (7.2)	0.17 (0.15, 0.21)	165 (2.4)	0.20 (0.17, 0.24)	
Urinary	47 (1.3)	0.03 (0.02, 0.06)	257 (3.8)	0.31 (0.27, 0.36)	
Male-specific	74 (2.2)	0.11 (0.08, 0.14)	1457 (21.5)	3.78 (3.55, 4.01)	
Lung	36 (1.0)	0.02 (0.02, 0.04)	211 (2.9)	0.24 (0.20, 0.28)	
Melanoma	628 (16.5)	0.39 (0.36, 0.43)	1030 (14.9)	1.22 (1.13, 1.32)	
Others	456 (13.1)	0.31 (0.28, 0.34)	576 (8.6)	0.70 (0.64, 0.78)	

CI, confidence interval; CRC, colorectal cancer; GI, gastrointestinal; PIR, family income-to-poverty ratio.

**Table 2:** Differences in trends in prevalence between long-term survivors of early-onset and late-onset cancer, 1997–2023.

Subgroups	Early-onset cancer, % (95% CI)		Changes		Late-onset cancer, % (95% CI)		Changes	
	1997	2023	%	AAPC (95% CI)	1997	2023	%	AAPC (95% CI)
Overall	1.62 (1.49, 1.78)	2.40 (2.20, 2.62)	48%	1.7 (1.4, 2.0)	5.41 (4.96, 5.89)	8.25 (7.76, 8.77)	52%	1.9 (1.7, 2.1)
Sex								
Men	0.83 (0.66, 1.05)	1.44 (1.22, 1.71)	129%	3.1 (2.5, 3.8)	5.76 (4.98, 6.64)	8.93 (8.20, 9.72)	70%	1.9 (1.6, 2.3)
Women	2.51 (2.25, 2.81)	3.31 (3.00, 3.66)	31%	1.3 (1.0, 1.6)	6.11 (5.42, 6.88)	7.65 (7.03, 8.32)	39%	1.7 (1.5, 2.0)
Age at interview, years								
25–49	1.20 (1.04, 1.39)	1.23 (1.01, 1.49)	3%	0.3 (−0.3, 0.8)	Not applicable	Not applicable		
50–69	2.52 (2.17, 2.91)	3.81 (3.39, 4.29)	51%	1.5 (1.0, 2.0)	2.61 (2.19, 3.10)	3.77 (3.30, 4.30)	44%	1.9 (1.4, 2.3)
70–85	1.70 (1.32, 2.19)	2.69 (2.32, 3.13)	58%	2.9 (2.2, 3.6)	9.21 (8.37, 10.12)	14.88 (13.93, 15.88)	62%	2.0 (1.8, 2.3)
Education								
Less than high school	1.61 (1.37, 2.06)	2.02 (1.45, 2.81)	20%	1.4 (0.7, 2.1)	5.57 (4.79, 6.46)	5.72 (4.58, 7.12)	3%	0.9 (0.4, 1.5)
High school	1.63 (1.37, 1.95)	2.15 (1.80, 2.57)	32%	1.2 (0.7, 1.7)	5.35 (4.53, 6.31)	7.78 (6.91. 8.74)	45%	1.9 (1.3, 2.4)
College or above	1.62 (1.41, 1.87)	2.55 (2.30, 2.81)	57%	2.0 (1.5, 2.4)	5.39 (4.60, 6.32)	9.08 (8.42, 9.78)	68%	2.5 (1.9, 3.1)
PIR								
<1.00	2.00 (1.56, 2.56)	2.09 (2.38, 4.00)	4%	1.2 (0.2, 2.2)	4.15 (3.09, 5.56)	6.76 (5.38, 8.47)	63%	1.1 (0.1, 2.0)
1.00–1.99	1.63 (1.30, 2.05)	2.68 (2.19, 3.29)	64%	1.4 (0.7, 2.1)	6.73 (5.68, 7.97)	7.01 (6.00, 8.18)	4%	1.0 (0.4, 1.7)
2.00–3.99	1.40 (1.14, 1.72)	2.23 (1.90, 2.61)	59%	1.8 (1.3, 2.3)	6.26 (5.31, 7.37)	9.46 (8.60, 10.39)	51%	1.7 (1.2, 2.3)
≥4.00	1.77 (1.46, 2.14)	2.47 (2.18, 2.80)	40%	1.4 (0.9, 1.9)	4.22 (3.38, 5.25)	8.23 (7.48, 9.06)	95%	3.2 (2.5, 3.9)
Race^a^								
Non-Hispanic White people	1.91 (1.70, 2.14)	3.06 (2.79, 3.36)	60%	2.0 (1.7, 2.4)	5.29 (4.73, 5.93)	9.48 (8.87, 10.14)	79%	2.4 (2.0, 2.9)
Non-Hispanic Black people	0.78 (0.54, 1.11)	1.60 (1.10, 2.32)	105%	2.2 (1.1, 3.3)	2.57 (1.77, 3.71)	7.01 (5.56, 8.80)	173%	3.3 (1.9, 4.8)
Hispanic	0.58 (0.36, 0.93)	1.07 (0.79, 1.44)	84%	3.3 (2.1, 4.5)	2.40 (1.48, 3.87)	4.03 (3.17, 5.11)	68%	2.0 (0.7, 3.3)
Others	0.80 (0.42, 1.52)	1.19 (0.76, 1.86)	49%	1.3 (−0.4, 3.1)	2.53 (1.08, 5.83)	4.03 (2.78, 5.81)	59%	2.6 (0.8, 4.4)
Survival, years								
5–9	0.44 (0.36, 0.53)	0.43 (0.35, 0.53)	-2%	−0.1 (−0.8, 0.6)	2.72 (2.40, 3.07)	3.25 (2.94, 3.60)	20%	0.8 (0.5, 1.5)
10–14	0.31 (0.25, 0.38)	0.39 (0.32, 0.49)	29%	0.9 (0.3, 1.6)	1.21 (1.00, 1.47)	2.38 (2.12, 2.67)	96%	2.3 (1.8, 2.7)
15–19	0.25 (0.19, 0.32)	0.36 (0.29, 0.46)	46%	2.2 (1.6, 2.9)	0.75 (0.58, 0.97)	1.31 (1.13, 1.51)	74%	3.3 (2.6, 4.0)
20–24	0.18 (0.13, 0.24)	0.37 (0.29, 0.46)	104%	2.1 (1.1, 3.2)	0.42 (0.31, 0.57)	0.80 (0.66, 0.97)	91%	3.2 (2.5, 3.9)
≥25	0.45 (0.37, 0.54)	0.85 (0.75, 0.97)	89%	3.0 (2.5, 3.5)	0.31 (0.22, 0.44)	0.51 (0.40, 0.65)	64%	3.2 (2.3, 4.1)
Cancer types								
Hematological	0.07 (0.04, 0.11)	0.14 (0.11, 0.20)	105%	3.0 (1.5, 4.4)	0.22 (0.14, 0.35)	0.42 (0.31, 0.56)	88%	2.5 (1.5, 3.4)
Breast	0.59 (0.48, 0.72)	1.00 (0.83, 1.19)	69%	2.4 (1.7, 3.0)	2.40 (2.07, 2.78)	3.39 (3.02, 3.81)	41%	1.7 (1.3, 2.1)
Gynecological	1.28 (1.11, 1.49)	1.07 (0.92, 1.26)	−16%	−0.4 (−0.9, 0.1)	1.13 (0.87, 1.48)	0.76 (0.56, 1.02)	−33%	0.1 (−0.7, 0.8)
CRC	0.08 (0.05, 0.12)	0.11 (0.06, 0.18)	33%	1.8 (0.2, 3.4)	0.62 (0.47, 0.82)	0.65 (0.53, 0.80)	5%	0 (−2.4, 2.5)
NonCRC GI	0.02 (0.01, 0.05)	0.03 (0.01. 0.06)	51%	1.4 (−1.1, 4.0)	0.10 (0.06, 0.17)	0.19 (0.12, 0.28)	87%	2.6 (1.2, 4.1)
Head and neck	0.03 (0.01, 0.07)	0.05 (0.03, 0.09)	95%	−0.3 (−2.2, 1.7)	0.21 (0.12, 0.34)	0.17 (0.11, 0.27)	−16%	−0.3 (−1.8, 1.2)
Thyroid	0.05 (0.03, 0.09)	0.16 (0.11, 0.23)	199%	4.1 (3.0, 5.2)	0.05 (0.02, 0.12)	0.25 (0.18, 0.35)	381%	6.0 (4.4, 7.6)
Urinary^b^	0.03 (0.01, 0.06)	0.02 (0.01, 0.04)	−39%	3.1 (0.9, 5.4)	0.23 (0.15, 0.37)	0.31 (0.22, 0.43)	30%	1.5 (0.2, 2.8)
Male-specific	0.12 (0.07, 0.20)	0.11 (0.66, 0.20)	−4%	−0.8 (−3.9, 2.5)	2.45 (2.00, 3.00)	4.03 (3.59, 4.53)	65%	2.2 (1.2, 3.2)
Lung	0.01 (0.00, 0.03)	0.02 (0.01, 0.05)	84%	0.8 (−1.6, 3.3)	0.25 (0.16, 0.38)	0.24 (0.17, 0.33)	−4%	0.4 (−0.3, 1.2)
Melanoma	0.13 (0.09, 0.18)	0.38 (0.30, 0.47)	195%	4.8 (3.7, 5.8)	0.29 (0.20, 0.43)	1.22 (1.04, 1.41)	316%	5.2 (0.5, 10.1)
Others	0.16 (0.12, 0.23)	0.36 (0.29, 0.46)	120%	3.4 (1.4, 5.4)	0.34 (0.24, 0.48)	0.69 (0.56, 0.85)	102%	4.9 (3.9, 5.9)

AAPC, average annual percentage change; CI, confidence interval; CRC, colorectal cancer; GI, gastrointestinal; PIR, family income-to-poverty ratio.

a The trend analyses for prevalence by race/ethnicity were from 1998 to 2023, as eligible data on race/ethnicity was not reported in 1997.

b The trend analyses for prevalence of urinary cancer were from 1998 to 2018, as information on kidney cancer was not reported from 2019.

### 3.3 Sex and age differences in prevalence and trends

Sex-specific analyses revealed women represented 72.7% of early-onset cancer survivors (prevalence 3.36, 95%CI: 3.20 to 3.52%), compared to 51.5% of late-onset cancer survivors (prevalence 7.91, 95%CI: 7.60 to 8.22%). Men showed lower prevalence of early-onset cancer survivors (1.36, 95%CI: 1.25 to 1.47%) but 6.3-fold higher prevalence of late-onset cancer survivors (8.54, 95%CI: 8.17 to 8.92%; Table 1). Temporal trend analyses found that from 1997 to 2023, the prevalence of early-onset cancer survivors increased by 129% in men compared to 31% in women, with AAPCs of 3.1 (2.5 to 3.8) and 1.3 (1.0 to 1.6), respectively. For prevalence of late-onset cancer survivors, both men and women showed a similar increasing trend, with AAPCs of 1.9 (1.6 to 2.3) and 1.7 (1.5 to 2.0), respectively (Figure 2; Table 2). When stratified by age groups, 55.2% of early-onset cancer survivors were aged 50–69 years, whereas 70.0% of late-onset cancer survivors were aged 70–85 years. Except for populations aged 25–49 years, which showed a stable trend, the prevalence of both early-onset and late-onset cancer survivors increased significantly from 1997 to 2023. In early-onset cancer survivors, the proportions were similar among survivors with different survival times, whereas late-onset cancer survivors were more likely to be 5–9 year survivors (Table 2).

### 3.4 Socioeconomic and racial disparities

Education level showed a clear gradient, with college-educated individuals accounting for 66.7% of early-onset cancer survivors and 61.8% of late-onset cancer survivors, with prevalence of 2.56% (95%CI:2.44 to 2.68%) and 8.73% (95%CI: 8.42 to 9.05%; Table 1). Compared with less than high school-educated populations (AAPC 0.9 95%CI: 0.4 to 1.5), the difference in increasing trends was only significant in late-onset cancer survivors in college-educated populations (AAPC 2.5 95%CI: 1.9 to 3.1; Table 2). Regarding PIR, the prevalence and their trends of early-onset cancer survivors were comparable among four PIR categories. In contrast, populations with higher PIR had higher prevalence and increasing trend, with the prevalence of 8.22% (95%CI: 7.88 to 8.58%) and AAPC of 3.2 (95%CI: 2.5 to 3.9) in populations with PIR higher than 4.00 (Tables 1, 2). Non-Hispanic White people had the highest prevalence of both early-onset (2.98, 95%CI: 2.85 to 3.11%) and late-onset (9.43, 95%CI: 9.14 to 9.73%) cancer survivors, whereas Hispanic populations showed the lowest prevalence of both early-onset (1.27, 95%CI: 1.07 to 1.50%) and late-onset (4.29, 95%CI: 3.75 to 4.90%) cancer survivors (Table 1). Prevalence of both early-onset and late-onset cancer survivors increased significantly from 1997 to 2023, although the differences were not significant among different races (Table 2).

### 3.5 Cancer-type specific prevalence and trends

**Figure 3:**
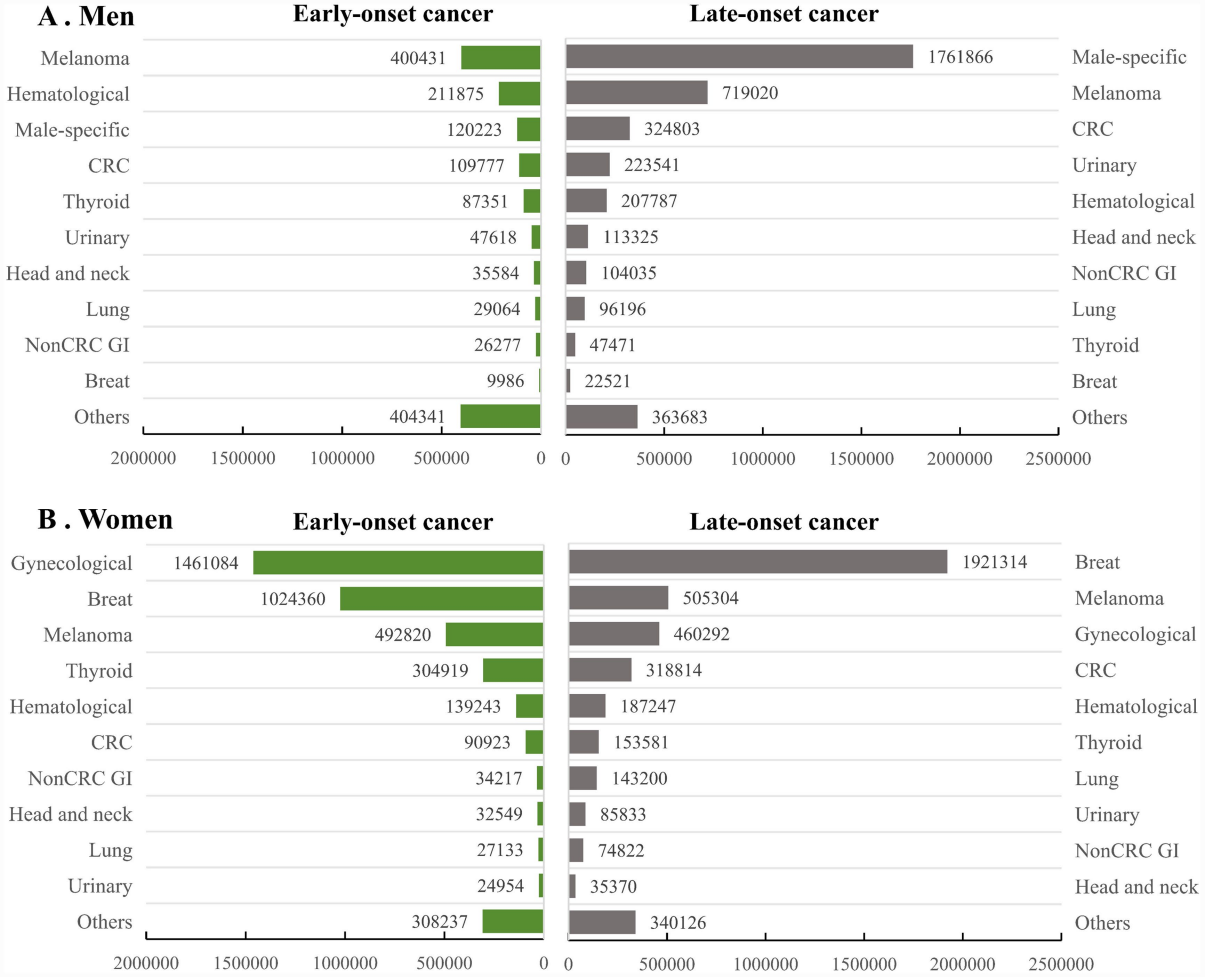
Prevalence of long-term cancer survivors among US male **(A)** and female **(B)** adults, 2019 to 2023. CRC, Colorectal cancer; GI, Gastrointestinal.

In male survivors, the most common early-onset cancers were melanoma (400431), hematological cancer (211875) and male-specific cancer (120223), whereas the most common late-onset cancers were male-specific cancer (1761866), melanoma (719020) and colorectal cancer (324803). In female survivors, the most common early-onset cancers were gynecological cancer (1461084), breast cancer (1024360) and melanoma (492820), whereas the most common late-onset cancers were breast cancer (1921314), melanoma (505304) and gynecological cancer (460293; Figure 3). The prevalence of the majority of late-onset cancers were higher than early-onset cancers, with male-specific cancers demonstrated the greatest difference (3.78% vs. 0.11%). In contrast, gynecological cancers were overrepresented in early-onset cancer survivors (26.9% vs. 5.6%; Table 1). From 1997 to 2023, the prevalence of survivors showed the most rapid increase in both early-onset melanoma (AAPC 4.8, 95%CI: 3.7 to 5.8) and late-onset melanoma (AAPC 5.2, 95%CI: 0.5 to 10.1). Similarly, the prevalence of survivors from early-onset and late-onset thyroid cancer, hematological cancer, breast cancer and urinary cancer demonstrated parallel acceleration, whereas the trends in prevalence of survivors from gynecological cancer, head and neck cancer, and lung cancer were stable. The significant increases in prevalence were found only in late-onset but not in early-onset non-colorectal gastrointestinal cancer and male-specific cancer. In contrast, the significant increase in prevalence was found only in early-onset but not in late-onset colorectal cancer (Table 2).

## 4 Discussion

This study provides a comprehensive analysis of prevalence trends among long-term cancer survivors, revealing critical differences between early-onset and late-onset cancer populations. Our findings illuminate the evolving epidemiology of cancer survivorship in the US over a 27-year period, with important implications for clinical practice and public health policy. The analysis of nationally representative NHIS data demonstrated several noteworthy patterns, including a persistent and significant increase in prevalence of both early-onset and late-onset long-term cancer survivors from 1997 to 2023, though absolute prevalence remained substantially higher among late-onset cancer survivors. This divergence was particularly pronounced in sex-stratified analyses, where men showed a remarkable increase in prevalence of early-onset cancer survivors compared to women, while both sexes exhibited parallel increases in late-onset cancer survivors. The socioeconomic gradient revealed college-educated individuals and those with higher PIR experienced the most rapid growth in prevalence, particularly among late-onset survivors. Cancer-type analyses identified distinct patterns, with gynecological cancers dominating early-onset survivorship while male-specific cancers showed the greatest disparity between onset groups.

Our study estimates approximately 13.63 million adult cancer survivors with ≥5-year survival in the US, slightly higher than the ACS/NCI 2022 estimate of 12.52 million (4). This discrepancy may stem from our inclusion of secondary and tertiary cancers as new survivor cases. Both findings underscore the substantial healthcare burden posed by long-term cancer survivors, particularly given their elevated risk of chronic diseases, secondary malignancies, and late mortality - risks that are especially pronounced among early-onset survivors (7, 15–17). Beyond estimating contemporary prevalence, we are the first to document increasing trends in long-term cancer survivorship, likely attributable to both rising incidence of certain cancers and therapeutic advancements (4). Importantly, treatment efficacy appears more influential than incidence alone in determining long-term survivor prevalence, as evidenced by the predominance and increasing trends of cancers with favorable prognoses (e.g., melanoma, thyroid cancer, breast cancer, and hematological malignancies) in both onset groups.

Our findings extend previous research by revealing striking sex-specific divergence in prevalence trends between early- and late-onset survivors. Men demonstrated significantly steeper increases in prevalence of early-onset cancer survivors compared to late-onset, while women showed slightly stronger trends for late-onset cancer survivorship. This pattern likely reflects complex interactions between biological factors, healthcare system characteristics, and social determinants. The accelerated rise in male early-onset survivorship may be driven by therapeutic advances for cancers disproportionately affecting younger men, including hematological, testicular, and colorectal cancers. Remarkable progress has been achieved in early-onset hematological cancers, benefiting from pediatric treatment innovations, while testicular cancer now boasts >95% 5-year survival due to cisplatin-based chemotherapy and refined surgical techniques (4, 15). Similarly, improved colorectal cancer therapies may increase survivorship among those diagnosed before age 50, a population showing rising incidence (18, 19). In contrast, female-predominant early-onset breast cancers already had relatively high survival rates by the 1990s, leaving less room for dramatic improvement (20, 21). Furthermore, uterine cancer is one of the few cancers for which no obvious advances in treatment have been achieved since the mid-1970s, leading to less improvement in survival of women with this disease (4). Additionally, female early-onset cancers are often diagnosed at more advanced stages with adverse pathological features - for instance, premenopausal women are more likely than postmenopausal women to present with triple-negative breast cancer, which carries poorer prognosis (22). Consequently, early-onset survivorship increases have been less pronounced than late-onset gains among women. The smaller sex disparity in late-onset survivorship aligns with the predominance of prostate cancer, breast cancer, and melanoma in older populations, where prostate cancer’s high prevalence and survival (98% 5-year rate) reflect widespread PSA screening and effective hormone therapies, while breast cancer survivorship gains plateaued after the 2000s as screening and treatment advances reached saturation (4). Furthermore, improved survivorship among younger men may also stem from reduced delays in diagnosis and treatment. Historically, men are less likely than women to seek preventive care, but awareness campaigns (e.g., for testicular self-exams) and shifts in healthcare utilization among younger generations could be narrowing this gap (23). Conversely, women’s lower trends in prevalence of early-onset cancer survivors may reflect persistent barriers in diagnosing gynecologic cancers (e.g., ovarian cancer’s nonspecific symptoms) (24). Beyond sociodemographic and healthcare-access factors, biological mechanisms may also contribute to the observed sex disparities. For instance, hormonal influences, such as estrogen and androgen signaling, could modulate tumorigenesis and treatment response of cancer (25). Additionally, emerging evidence suggests that tumor genomics and immune microenvironment features may differ by sex and age at diagnosis, potentially affecting long-term outcomes (26, 27). These biological factors likely interact with social determinants to shape survivorship trajectories, though further mechanistic studies are needed to elucidate their precise roles.

The socioeconomic gradients we identified are consistent with findings from previous studies that socioeconomic deprivation increases all-cause and cardiovascular disease mortality in the general population and cancer survivors (28, 29). However, our study uniquely reveals that these disparities are more pronounced among late-onset cancer survivors, suggesting that socioeconomic advantages compound with age, enabling better access to early detection and long-term care. Similarly, racial disparities in our study mirror those reported in SEER data, where non-Hispanic White people had higher survivorship rates compared to Hispanic populations (4, 30). Yet, our analysis adds nuance by showing that these disparities persist across both early- and late-onset groups, albeit without significant differences in trend magnitudes. Socioeconomic disparities in survivorship can be attributed to compounded advantages: higher-income individuals not only benefit from better healthcare access but also from superior management of chronic post-treatment conditions, which is particularly critical for late-onset survivors, who often contend with multimorbidity (31–33).

These findings carry several urgent implications for clinical and public health practice. The distinct comorbidity profiles between early- and late-onset cancer survivors demand differentiated care models. Early-onset cancer survivors may benefit from enhanced screening for premature chronic diseases and fertility preservation counseling, given their prolonged life expectancy and reproductive concerns (34). For example, breast cancer survivors under 50 face elevated risks of osteoporosis and cardiovascular disease due to hormonal therapies, necessitating early intervention (35, 36). Late-onset survivors, on the other hand, require integrated oncology-geriatrics approaches to manage interactions between cancer sequelae and age-related conditions (37). The high prevalence of prostate cancer among older men, for instance, underscores the need for personalized surveillance strategies to balance active treatment with quality-of-life considerations (38). The concentration of survivorship gains among populations with better socioeconomic status calls for policies to expand access to care in underserved communities. For example, Medicaid expansion and community-based survivorship programs could mitigate disparities, particularly for late-onset cancers where gaps are most pronounced (39, 40). Cancer-type-specific trends further suggest the need for targeted prevention strategies. The rising prevalence of melanoma among older adults highlights the importance of sun protection education beyond childhood, while the low prevalence and stability of survivorship trends of some cancers that have high incidence, such as lung cancer, underscores the ongoing need for advances in treatment (41–43).

Several limitations warrant consideration in interpreting these results. Self-reported cancer history may introduce misclassification, though NHIS validation studies suggest high specificity for major cancers. The cross-sectional design precludes causal inferences about factors driving prevalence changes, and the lack of clinical details like stage at diagnosis and treatment modalities may mask important subgroup variations. The 5-year survival threshold, while clinically meaningful, may not fully capture differences in very long-term outcomes between onset groups. In addition, our findings regarding the compounding of socioeconomic disparities with age must be interpreted within the context of the U. S. healthcare system, which ties insurance coverage and care access heavily to employment and income. In countries with robust universal healthcare, the socioeconomic gradient in survivorship might manifest differently. Future research should incorporate tumor registry linkages to validate self-reports and enable stage-specific analyses, examine the interplay between comorbid conditions and survivorship patterns using electronic health record data, and investigate care utilization differences between onset groups to identify modifiable service gaps. International comparisons could help disentangle healthcare system effects from biological factors, and prediction models integrating both cancer-related and sociodemographic variables could identify high-risk survivorship subgroups.

## 5 Conclusion

This 27-year analysis reveals dynamic, onset-specific trajectories in cancer survivorship prevalence, shaped by complex interactions between biological factors, healthcare advances and access, and social determinants. The diverging patterns between early and late-onset cancer survivors underscore that “cancer survivor” is not a monolithic category but rather encompasses populations with distinct clinical needs and health system requirements. Our findings argue for a paradigm shift in survivorship care - from uniform approaches to precision strategies accounting for diagnosis age, sex, socioeconomic context, and cancer type. As survivorship continues growing globally, these results provide an evidence base for targeted resource allocation and policy development to ensure equitable long-term outcomes across all survivor populations. The persistent disparities identified serve as both a metric of progress and a reminder of the work remaining to achieve comprehensive, patient-centered survivorship care.

## Data Availability

The original contributions presented in the study are included in the article/supplementary material, further inquiries can be directed to the corresponding author.
